# Epigenetic Aspects and Prospects in Autoimmune Hepatitis

**DOI:** 10.3389/fimmu.2022.921765

**Published:** 2022-06-30

**Authors:** Albert J. Czaja

**Affiliations:** Retired, Rochester, MN, United States

**Keywords:** autoimmune, hepatitis, epigenome, chromatin modifications, micro-ribonucleic acids, treatment

## Abstract

The observed risk of autoimmune hepatitis exceeds its genetic risk, and epigenetic factors that alter gene expression without changing nucleotide sequence may help explain the disparity. Key objectives of this review are to describe the epigenetic modifications that affect gene expression, discuss how they can affect autoimmune hepatitis, and indicate prospects for improved management. Multiple hypo-methylated genes have been described in the CD4^+^ and CD19^+^ T lymphocytes of patients with autoimmune hepatitis, and the circulating micro-ribonucleic acids, miR-21 and miR-122, have correlated with laboratory and histological features of liver inflammation. Both epigenetic agents have also correlated inversely with the stage of liver fibrosis. The reduced hepatic concentration of miR-122 in cirrhosis suggests that its deficiency may de-repress the pro-fibrotic *prolyl-4-hydroxylase subunit alpha-1 gene*. Conversely, miR-155 is over-expressed in the liver tissue of patients with autoimmune hepatitis, and it may signify active immune-mediated liver injury. Different epigenetic findings have been described in diverse autoimmune and non-autoimmune liver diseases, and these changes may have disease-specificity. They may also be responses to environmental cues or heritable adaptations that distinguish the diseases. Advances in epigenetic editing and methods for blocking micro-ribonucleic acids have improved opportunities to prove causality and develop site-specific, therapeutic interventions. In conclusion, the role of epigenetics in affecting the risk, clinical phenotype, and outcome of autoimmune hepatitis is under-evaluated. Full definition of the epigenome of autoimmune hepatitis promises to enhance understanding of pathogenic mechanisms and satisfy the unmet clinical need to improve therapy for refractory disease.

## 1 Introduction

Autoimmune hepatitis has genetic risk factors within and outside the major histocompatibility complex (MHC) (1, 2). The genetic risk factors within the MHC affect mainly the predisposition for autoimmune hepatitis. The susceptibility alleles reside on the *HLA-DRB1 gene* where they can vary in association with ethnicity and age (3–9). The genetic risk factors outside the MHC are less established. They are mainly polymorphisms or point mutations that may affect individual pathways within the immune response (cytokine milieu, lymphocyte activation, and cell migration) (1, 2, 10–18). The major risk-laden loci are present in approximately 50% of patients with autoimmune hepatitis (19), and they do not explain the observed risk of the disease (19–21).

Epigenetics is a burgeoning science that describes molecular modifications and mechanisms that can modulate gene activity without altering the nucleotide sequence of deoxyribonucleic acid (DNA) (22–26). The epigenetic changes have cell type specificity and stability through cell replication (27), and they have been heritable in diverse experimental models (25, 28). Key epigenetic modifications have been described in the nuclear chromatin that can affect gene transcription (29–31), and small non-coding ribonucleic acids are epigenetic agents that can affect translation of the gene product (32, 33). The epigenetic modifications may be induced by environmental cues (34–37), and they have a durability that may contribute to a transgenerational inheritance through the germline (25, 28, 37, 38). Furthermore, the epigenetic changes are modifiable, reversible, and amenable to therapeutic intervention (19, 39–43).

Epigenetics may explain the difference between the genetic risk and observed risk of autoimmune hepatitis, and it may account for individual variations in clinical phenotype and outcome that cannot be explained by the MHC, genetic polymorphisms, or point mutations (39, 44–47). Chromosomal regions may undergo structural adaptations in response to environmental cues that alter DNA transcription (22, 38), and non-coding ribonucleic acids, especially micro-ribonucleic acids (miRNAs), may induce degradation or translational repression of messenger ribonucleic acids (mRNAs) (48–53).

Salient epigenetic effects have already been identified in experimental models and patients with diverse liver diseases, including alcoholic steatohepatitis (54, 55), non-alcoholic fatty liver disease (NAFLD) (56–58), primary biliary cholangitis (PBC) (59–61), primary sclerosing cholangitis (PSC) (62–64), cholangiocarcinoma (62, 65–67), hepatocellular cancer (68, 69), and autoimmune hepatitis (21, 70, 71). They have also been implicated in various non-liver diseases, including systemic lupus erythematosus (SLE) (72, 73), rheumatoid arthritis (74, 75), systemic sclerosis (76, 77), diverse neuro-degenerative diseases (78), and various cancers (79–82). Investigations of the epigenetic modifications affecting gene expression in autoimmune hepatitis may improve its management and satisfy an unmet clinical need for more effective therapy of refractory disease (83–85).

The goals of this review are to describe the epigenetic modifications that affect gene expression, examine transgenerational inheritance of epigenetic marks, present the key epigenetic changes in autoimmune hepatitis and other liver diseases, and indicate the prospects that epigenetics will enhance understanding of pathogenic pathways and treatment options in autoimmune hepatitis.

## 2 Methods

Abstracts were identified in PubMed using the search words “Epigenetic changes in liver disease,” “Epigenetic changes in autoimmune hepatitis”, “microRNAs in liver disease”, and “microRNAs in autoimmune hepatitis”. Selected full-length articles constituted the primary bibliography. Selected references cited in the primary sources constituted a secondary bibliography, and a tertiary bibliography was developed from references cited in the secondary bibliography. Several hundred abstracts were reviewed, and the number of full-length articles that were examined was 205.

## 3 Epigenetic Modulation of Gene Transcription

**Figure 1:**
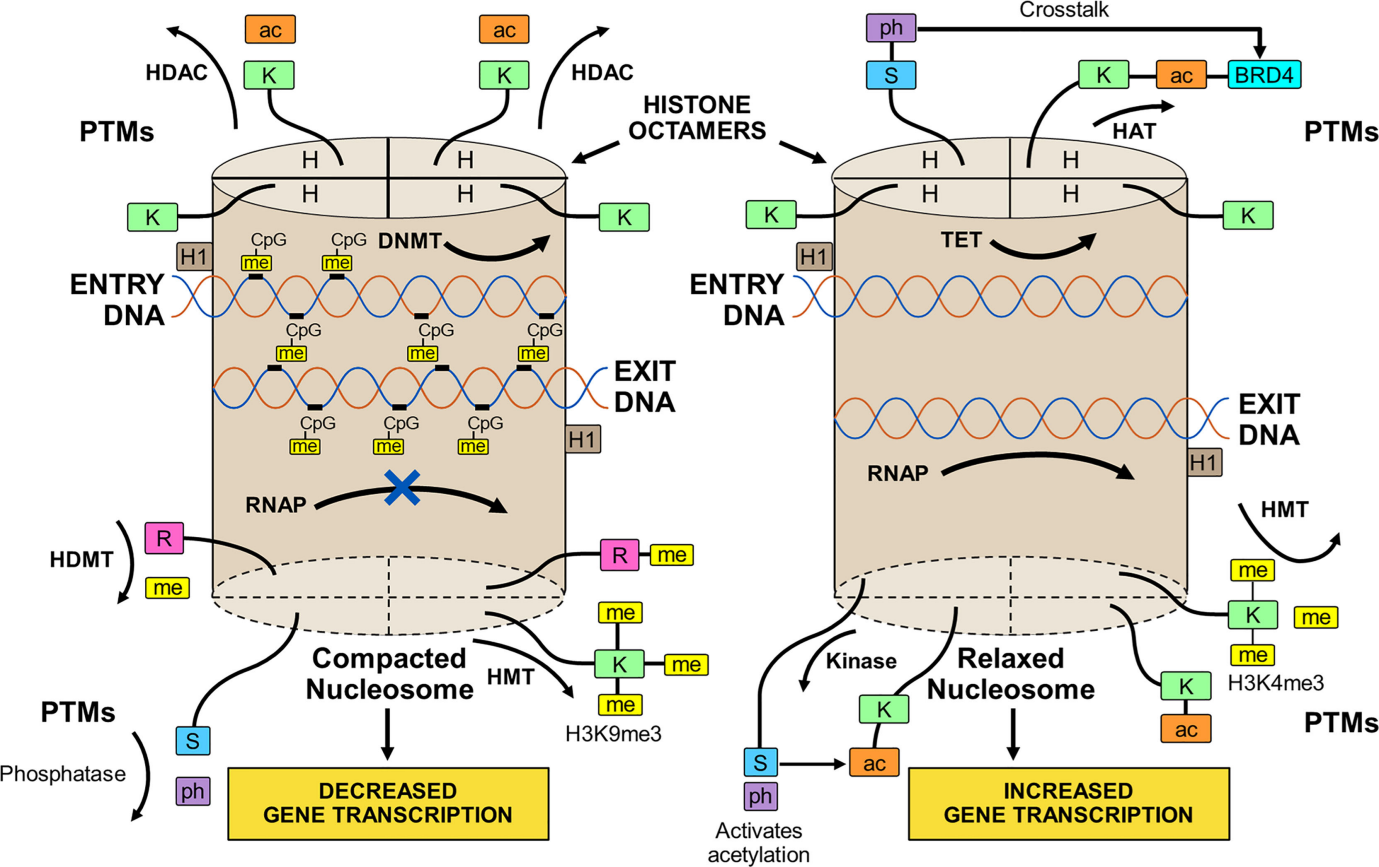
Compacted and relaxed nucleosomes. Nucleosomes consist of two copies of four different histones (H) arranged as a histone octamer and double-stranded deoxyribonucleic acid (DNA) wrapped 1.65 times around each octamer. The entry and exit of the DNA from the nucleosome is secured by a linker histone (H1). Each core histone within the octamer has an N-terminal tail that can undergo post-translational modifications (PTMs) by the attachment of methyl (me), acetyl (ac), or phosphate (ph) groups to a particular amino acid in the histone tail. Lysine (K), serine (S), or arginine (R) are among other amino acids that can serve as attachment sites. The PTMs are orchestrated by various enzymes. Methylation of the histone tail is catalyzed by histone methyltransferase (HMT); acetylation is catalyzed by histone acetyltransferase (HAT); and phosphorylation is catalyzed by kinases. The PTMs can be reversed by enzymes that dissociate the appended groups from the amino acid residues. Acetylation is reversed by histone deacetylase (HDAC); methylation is reversed by histone demethylase (HDMT); and phosphorylation is reversed by phosphatases. Histone acetylation relaxes the nucleosome and promotes gene transcription, and histone de-acetylation compacts the nucleosome (heterochromatin) and represses gene transcription. Histone methylation can decrease (H3K9me3) or increase (H3K4me3) transcription depending on the methylation site and other variables. Histone phosphorylation can recruit other molecules, such as bromo-domain-containing protein 4 (BRD4), to the acetylation site (crosstalk) and promote gene transcription. DNA can be methylated by DNA methyltransferase (DNMT) or de-methylated by ten-eleven translocation methylcytosine dioxygenase (TET). DNA methylation is restricted to sites in which cytosine (C) is separated from guanine (G) by a phosphate (p). Methylated DNA is compacted and transcription factors have limited access to transcription sites. Ribonucleic acid polymerase (RNAP) is prevented (X) from copying the nucleotide sequence, and gene transcription is decreased. De-methylated DNA is relaxed; RNAP can open the double-stranded DNA; and gene transcription is increased.

The transcriptional activity of genes occurs within chromatin (86). Chromatin is composed of histones arranged in octamers and double-stranded DNA that makes 1.65 turns around each octamer (38, 39, 86, 87) (**Figure 1**). Two copies of four core histones (H2A, H2B, H3, and H4) comprise the octamer (38, 86–88), and each DNA-enwrapped octamer constitutes a nucleosome (89). The nucleosomes are linked by a short DNA sequence of 60 base pairs, and the beaded filament is condensed and packaged in the nucleus as chromatin (86). A histone linker molecule maintains proper packaging of the DNA by binding to the site of DNA entry and exit from each nucleosome (86, 87, 90).

### 3.1 Impact of DNA Methylation on Gene Transcription

The methylated state of the DNA (39, 91–93) influences transcriptional activity within the nucleosome. Modifications in the chromatin structure can alter access and binding of transcription factors to the enhancer/promoter sequences of the DNA that are pivotal for transcription (94, 95) (**Figure 1**). The inability of RNA polymerase (RNAP) to access the DNA binding site can prevent opening of the double-stranded DNA and copying of the nucleotide sequence (39, 92).

DNA methylation occurs at a site in which a cytosine nucleotide (C) is separated from a guanine nucleotide (G) by a phosphate molecule (p) (46) (**Table 1**). Methylation of the cytosine in the CpG dinucleotide to 5-methylcytosine is mediated by DNA methyltransferases (DNMTs), and the methylation inhibits the binding of transcription factors to the DNA (91, 92) (**Figure 1**). It can also alter chromatin structure by attracting proteins that bind to the methylated cytosine (46, 92). The net effect of DNA methylation is to repress transcriptional activity and silence gene expression (39).

**Table 1:** Epigenetic properties and effects on gene transcription.

Epigenetic Mark	Epigenetic Properties	Epigenetic Effects on Transcription
DNA methylation	CpG methylated at cystosine (46) DNMT catalyzes 5-methylcytosine (91) Attached proteins alter chromatin (92) Transcription factors denied access (94) RNAP unable to copy DNA (39, 92)	Transcriptional activity repressed (39)
DNA de-methylation	Cytosine demethylation by TETs (96, 97)	Transcriptional activity increased (39)
Histone acetylation	Lysine on histone tail acetylated (39, 86) Acetyl group from acetyl-CoA (98, 99) HATs mediate acetyl group transfer (78) Histone-DNA charges less (86) Chromatin structure relaxed (39, 86)	Transcriptional activity increased (86)
Histone de-acetylation	HDACs hydrolyze acetyl group (86) Heterochromatin formed (100)	Transcriptional activity repressed (86)
Histone methylation	Methyl groups from SAM (101–105) Added to lysine or arginine (101, 103, 105) HMTs catalyze methyl transfer (86, 104) No effect on charge of histone tail (86) Recruited molecules affect gene (106–110)	Unpredictable transcriptional effect (86) Varies by site, number, pattern (86, 101) H3K4me3 activates transcription (111) H3K9me3 silences transcription (106)
Histone de-methylation	HDMTs remove methyl groups (104, 112) Counterbalances HMTs (86)	Unpredictable transcriptional effect (86) Responds to changing conditions (86)
Histone phosphorylation	Phosphates from ATP by kinases (113) Affects serine, threonine, tyrosine (113) Adds negative charge to histone (113) Compacts or relaxes chromatin (114, 115) Reversed by phosphatases (113)	Variable, context-related effects (114) DNA damage response (114, 116, 117) Interacts with acetylated residues (114)

ATP, adenosine triphosphate; CpG, cytosine-phosphate-guanine dinucleotide; DNA, deoxyribonucleic acid; DNMTs, DNA methyltransferases; H3K4me3, trimethylation of histone H3 at lysine 4; H3K9me3, trimethylation of H3 at lysine 9; HATs, histone acetyltransferases; HDACs, histone deacetylases; HDMTs, histone demethylases; HMTs, histone methyltransferases; RNAP, RNA polymerase; SAM, S-adenosylmethionine; TETs, ten-eleven translocation enzymes. Numbers in parentheses are references.

Ten-eleven translocation methylcytosine dioxygenase (TET) enzymes mediate the oxidation of the methylated cytosine to 5-hydroxymethylcytosine (39, 96, 97, 118, 119) (**Table 1**). This product can then undergo additional processing and demethylation by thymine-DNA-glycosylase and excision repair (118, 120, 121). The restoration of cytosine to its unmodified state can de-repress transcriptional activity and promote gene expression (**Figure 1**). The counter effects of DNMTs and TET enzymes on DNA methylation constitute a homeostatic mechanism that can respond to diverse stimuli, be disrupted in disease states, and be manipulated by therapeutic interventions (39, 122).

### 3.2 Impact of Histone Modifications on Gene Transcription

The N-terminal tail of the core histones can undergo multiple post-translational modifications (PTMs) that include acetylation, methylation, and phosphorylation (39, 86, 113, 123–126) (**Table 1**). The PTMs can alter the chemical structure, charge, and configuration of the histones, and the cumulative effect of multiple histone modifications can determine the transcriptional activity of the DNA (127) (**Figure 1**). PTMs also influence the cellular repair response to DNA injury (128). The modification of histones is a dynamic process that can preserve the integrity of the genome (129) and modulate transcriptional activity to maintain biological homeostasis (39, 86).

#### 3.2.1 Histone Acetylation

The transfer of an acetyl group from acetyl-coenzyme A (acetyl-CoA) to a lysine residue on the histone tail constitutes histone acetylation, and the process is mediated by the histone acetyltransferases (HATs) (39, 78, 86, 98, 99) (**Table 1**). Histone acetylation can promote transcriptional activity by neutralizing differences in charge between the positively charged histones and the negatively charged DNA. The relaxed chromatin can promote transcriptional activity (39, 86) (**Figure 1**). Histone deacetylases (HDACs) can reverse the acetylation process by hydrolyzing the acetyl group on the lysine residue, compacting the chromatin into heterochromatin, and repressing transcriptional activity of the DNA (78, 86, 99, 100).

#### 3.2.2 Histone Methylation

The transfer of methyl groups from S-adenosylmethionine (SAM) to lysine or arginine residues on the histone tail constitutes histone methylation (101–105), and the methylation process is mediated by histone methyltransferases (HMTs) (86, 104, 128, 130) (**Table 1**). The impact of histone methylation on DNA transcription is less predictable than histone acetylation, and it varies by methylation site (lysine versus arginine), number of methylations (mono-, di-, or tri-methylation) and pattern of methylation (symmetric versus asymmetric) (101, 103, 104, 128). Trimethylation of histone H3 at lysine 4 (H3K4me3) is the start site of transcription for most active genes (111, 128, 131, 132) (**Figure 1**), whereas trimethylation of H3 at lysine 9 (H3K9me3) is associated with heterochromatin and gene silencing (106, 128, 132).

Histone methylation does not affect the charge of the histone tail, and the impact of histone methylation on transcriptional activity relates mainly to the effects of molecules recruited to the methylated state and the sequence of adjacent amino acids (86, 107). Lysine methylation attracts diverse proteins mainly with chromo-domains that can modify chromatin structure and affect DNA transcription (108–110). Histone demethylases (HDMTs) can reverse the methylated PTM by removing methyl groups from the histone tails (86, 104, 112). The balance between HMTs and HDMTs is another homeostatic mechanism by which the genome can respond to changing conditions.

#### 3.2.3 Histone Phosphorylation

Histone phosphorylation is a dynamic process affecting serine, threonine, and tyrosine residues in the N-terminal tail of the core histones (113) (**Table 1**). Kinases transfer phosphate groups from adenosine triphosphate (ATP) to the amino acid residues. Phosphorylation adds a negative charge to the histone, and the change in charge can remodel the chromatin. The phosphorylation process can be reversed by phosphatases that catalyze the hydrolysis and removal of the phosphate group (113) (**Figure 1**).

Histone phosphorylation occurs rapidly after DNA damage, and it is involved in the DNA damage response (DDR) (114, 116, 117) (**Table 1**). Phosphorylated histone residues are also associated with gene expression, including proto-oncogenes (133–135), and they can interrelate with histone residues that are acetylated to activate DNA transcription (114, 136, 137) (**Figure 1**). Phosphorylation of serine 10 at histone H3 (H3S10ph) activates DNA transcription by triggering acetylation of lysine 16 at histone 4 (H4K16ac) (138). The crosstalk between histone PTMs recruits bromo-domain-containing protein 4 (BRD4) to the nucleosome where it can bind to the acetylated lysine residue and promote DNA transcription (138–140).

The phosphorylation of histone is a rapidly changing process that can have contradictory effects depending on the context of the microenvironment (114). Histone phosphorylation is associated with chromatin compaction during mitosis and meiosis, but it can also be associated with chromatin relaxation under other circumstances (114, 115, 141). Therapeutic efforts to modulate histone phosphorylation must recognize the dynamic, interactive, labile, and context-dependent nature of the PTM.

## 4 Epigenetic Modulation of Gene Translation

**Figure 2:**
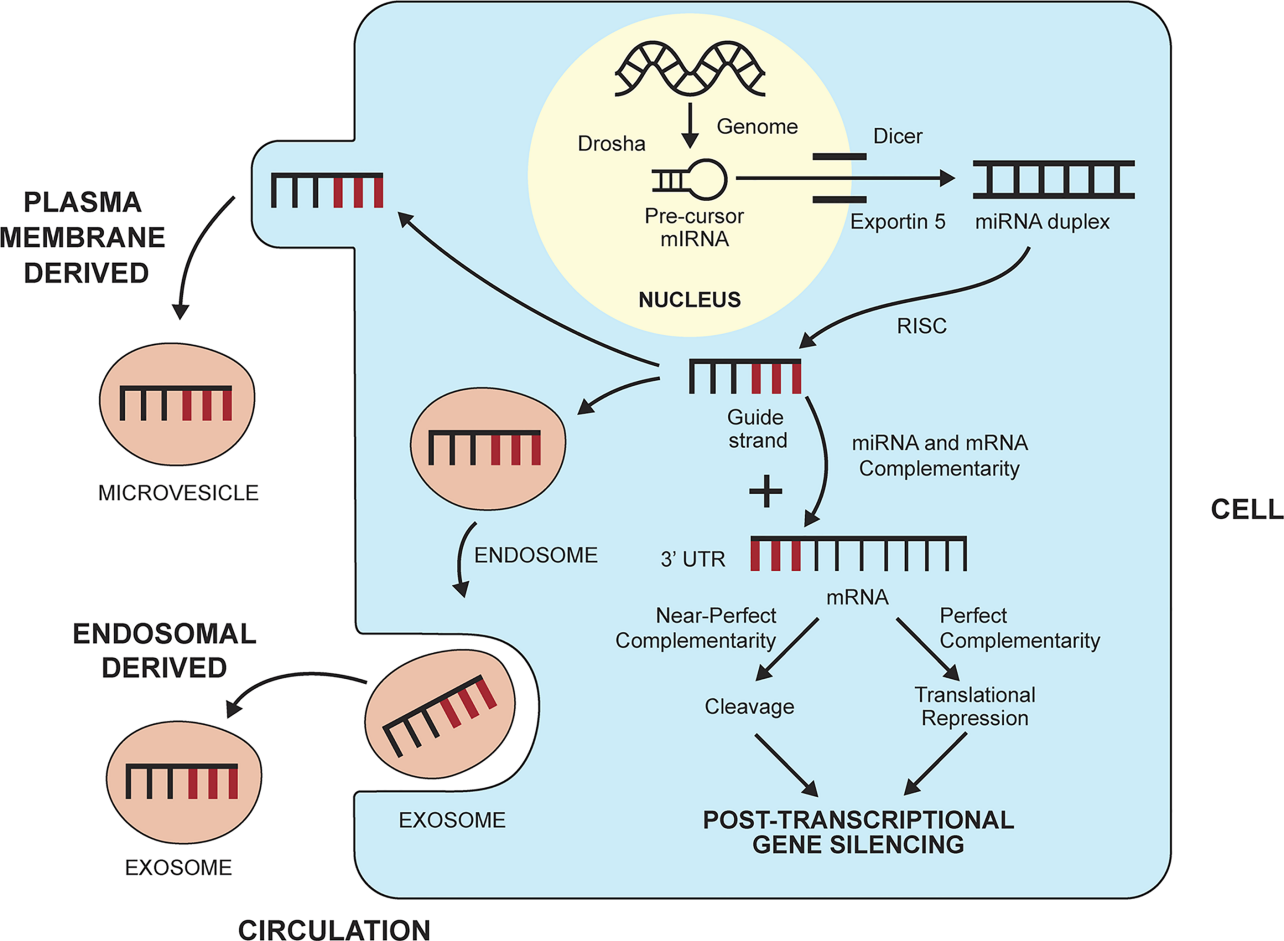
Biogenesis and gene silencing action of micro-ribonucleic acids (miRNAs). MiRNAs are derived from the cell genome and processed within the nucleus by the ribonuclease III enzyme, Drosha, into pre-cursor miRNA. The precursor miRNA is transported to the cytoplasm by exportin 5 and processed further by the ribonuclease II enzyme, Dicer, to a miRNA duplex. The duplex is processed in a RNA-induced silencing complex (RISC), and the strand with less stable 5’ end is selected as the guide strand. The guide strand probes for complementary base pairs (bold lines) in the 3’ untranslated region (3’UTR) of messenger RNA (mRNA). The degree of complementarity between the guide strand and the mRNA determines if the mRNA will undergo cleavage by endonucleases (perfect complementarity) or translational repression (near perfect complementarity). Either fate induces post-transcriptional gene silencing. MiRNAs can leave the cell and enter the circulation by forming a plasma membrane-derived microvesicle or an endosomal-derived exosome.

MiRNAs are a subgroup of non-coding RNAs that by definition do not encode protein (142) (**Table 2**). They constitute a functional minority of non-coding RNAs (143), and they are members of a class that includes small interfering RNAs (siRNAs) (144) and Piwi-interacting RNAs (piRNAs) (145, 146). MiRNAs are small (21-25 nucleotides), natural, genomic products that have multiple functions within their cell of origin (48, 50). They are present in the nucleus, nucleolus, and mitochondria where they can influence the intracellular processes of DNA transcription, repair, and splicing (147, 148, 170, 171). They can also silence the expression of genes that encode protein by preventing the translation of mRNA into a protein product (48, 50, 51) (**Figure 2**). MiRNAs are key epigenetic agents that act primarily outside the chromatin to degrade mRNA within the cytoplasm or otherwise repress its translation.

**Table 2:** Epigenetic Properties and Effects of Micro-Ribonucleic Acids.

miRNA Properties	miRNA Actions	miRNA Effects
Non-coding RNA (142–146) •Small (21-25 nt) (48, 50) •Derived from genome (48) •Present in organelles (147)	Prevents mRNA translation (48) Regulates cell processes (148)	Maintains cell homeostasis (147) Responds to changing context (147)
Circulatory component (149) •Vesicular transport (150, 151)	Cell-to-cell communication (149) May affect other cell function (152)	Correlates with inflammation (70)
Diverse cell origins (153) •>500 in humans (154) •Multiple cell origins (153) •One targets many genes (155) •Many target same gene (156)	Critical physiological effects (157) Context-cell dependent (157, 158) Affects protein-encoding genes (50) Specific for certain cell lines (159)	Variable disease specificity (153) Many are disease-irrelevant (153) May have distinctive patterns (70) Associated with diseases (71, 160, 161)
Complex biogenesis (149, 162) •Nuclear origin (149) •Enzymatic processing (163) •Exported to cytoplasm (164) •Processed in RISC (51) •Guide strand selected (165) •Incorporated into RLC (165) •Non-canonical pathways (166–168)	Guide strand seeks mRNA (155) Binds 3’ UTR of mRNA (51, 155)	Depends on complementarity (169) Promotes mRNA degradation (169) Represses mRNA translation (149) Gene silencing (169)

mRNA, messenger ribonucleic acid; miRNA, micro-ribonucleic acid; nt, nucleotides; RNA, ribonucleic acid; RISC, RNA-induced silencing complex; RLC, RISC-loading complex; 3’ UTR, 3’ untranslated region of mRNA. Numbers in parentheses are references.

MiRNAs can enter the circulation within vesicles that develop from the endosomal compartment (exosomes) or separate from the plasma membrane (microvesicles, apoptotic bodies) (150, 151) (**Figure 2**). Circulating miRNAs have the potential to engage in cell-to-cell communication and affect the function of other cells, albeit their role in this capacity remains obscure (149, 152) (**Table 2**). Despite this limitation, circulating levels of miRNAs have been measured and correlated with the inflammatory activity of diverse diseases, including autoimmune hepatitis (70).

The number of miRNAs in humans has been estimated by a manually curated miRNA database as over 500 (154) (**Table 2**). Multiple miRNAs can regulate the expression of a single gene, and a single miRNA can influence multiple genes (155, 156). Diverse cell types produce miRNAs, and tissue and disease specificity can be difficult to demonstrate (153). Critical physiological and pathological effects have been ascribed to single miRNA-mRNA interactions that are context-dependent (157, 158), and certain miRNAs have been highly specific for individual cell lines (159). Preferential expression of particular miRNAs has been recognized in diverse diseases, including chronic liver disease (70, 71, 160, 161).

### 4.1 Biogenesis and Regulatory Actions of MiRNAs

MiRNAs originate in the nucleus as double-stranded RNA molecules that are encoded by the genome as primary miRNAs (149, 162) (**Table 2**). The primary miRNAs are then modified in the nucleus by a microprocessor complex containing the ribonuclease III enzyme, Drosha, to precursor miRNAs (149, 163, 172, 173) (**Figure 2**). The precursor miRNAs are exported to the cytoplasm by exportin 5 where the ribonuclease II enzyme, Dicer, modifies the precursor molecules further to form mature miRNA duplexes (164, 174). The duplexes are processed in a RNA-induced silencing complex (RISC) within the cytoplasm (51), and the strand with the less stable 5’ end is selected for incorporation in the RISC-loading complex (RLC) as the guide strand (165). The other strand (passenger strand) is degraded by endonucleases (175).

The guide strand probes for complementary base pairs in the 3’ untranslated region (3’ UTR) of mRNAs in the cytoplasm (**Figure 2**). The “seed region” that identifies complementarity in the mRNA may consist of only 2-7 bases (51, 155). Near perfect complementarity between the miRNA and the mRNA triggers degradation of the mRNA by endonucleases and complete gene silencing (169) (**Table 2**). More commonly, the complementarity is less complete, and the miRNA mainly disrupts the translation of mRNA without triggering its degradation (translational repression) (51, 149). MiRNAs can also develop along non-canonical pathways that do not involve Drosha or Dicer (166–168). The biological functions of these miRNAs are uncertain in humans.

## 5 Transgenerational Inheritance of Epigenetic Marks

The DNA sequence and the epigenome are replicated during cell mitosis (25, 27), and DNA methylation (176), histone PTMs (38), and miRNAs (177) can be transmitted in the germline of mammals. Extensive re-programming of the epigenetic information occurs during gametogenesis and after fertilization, and transgenerational inheritance requires re-assembly or reconstruction of the epigenetic marks. DNA methylation and histone modifications can be re-assembled after mitosis (replicative transmission) or the epigenetic changes can be reconstructed in the germline by another inherited signal (reconstructive transmission) (28). Non-coding RNAs are templates that are pivotal to the reconstructive process, and they can be transmitted to the next generation in oocytes and sperm (28, 178, 179). Transgenerational inheritance requires proof that the original epigenetic signal is successfully transmitted and that heritability extends beyond the second generation.

The transmitted epigenetic changes may reflect environmental adaptations made by the parent and transmitted to the offspring through the germline (28, 35, 180, 181). The offspring of male mice who have been fed a low-protein diet inherit epigenetic marks that affect the *peroxisome proliferator-activated receptor alpha (PPARA) gene* which regulates lipid and cholesterol metabolism (180). The heritable epigenetic changes may also re-program responses to disease (182). Two generations of offspring from male rats with a history of liver fibrosis have inherited a resistance to hepatic fibrosis manifested by impaired differentiation of hepatic myofibroblasts, increased expression of the anti-fibrotic peroxisome proliferator-activated receptor-gamma (PPAR-γ) protein, and decreased production of the pro-fibrotic transforming growth factor beta 1 (TGF-β1) cytokine (182).

The demonstration of heritable epigenetic marks has been difficult to establish in humans because of confounding genetic, cultural, and environmental factors (37, 183), and heritability has been eliminated from the definition of epigenetics (22). Epigenetic changes within an individual may be acquired by external pressures (diet, lifestyle, toxic exposures) (184–187) or by intrinsic instability of the epigenome through successive cell divisions (“epigenetic drift”) (188–192). Shared changes in the somatic epigenome of individuals in the same environment does not connote heritability unless expressed in the germline (sperm or egg) (25). Furthermore, the epigenetic marks in individuals with genetic identity cannot be assumed to be inherited. Genetically identical monozygotic twins may acquire epigenetic changes that are distributed throughout the genome and related to the commonality or diversity of their environment (187).

Family studies assessing discordant and concordant phenotypes have demonstrated the complexity of distinguishing inherited and acquired determinants. Fatty liver occurs in 17% of siblings and 37% of parents of overweight children (193). The severity of hepatic steatosis in the family members strongly correlates with body mass index (BMI) (193). Complete hereditability for fatty liver is evident after adjustments for age, gender, race, and BMI, but the phenotypic expression of the inherited risk probably relates to family attitudes about diet and exercise (193). Similarly, heritable miRNAs for NAFLD (miR-331-3p and miR-30c) have been demonstrated in monozygotic and dizygotic twins, but most of the 21 miRNAs that have distinguished the twins with and without NAFLD have not been inherited (194). Although transgenerational inheritance of epigenetic marks has been demonstrated in experimental models (28, 180, 182) and humans (26, 37, 194), its impact on the occurrence of an individual disease is unsettled (35). Large longitudinal studies over several generations are necessary to establish the heritability of epigenetic changes in particular human diseases, and they would require concurrent analyses of the genome and epigenome (37, 183, 195).

## 6 Epigenetic Changes in Autoimmune Hepatitis

Investigations of the epigenetic changes in patients with autoimmune hepatitis have been limited, and they have focused mainly on DNA methylation patterns in circulating and liver-infiltrating lymphocytes (21) and on the profile of circulating miRNAs (70).

### 6.1 DNA Methylation Patterns

Most genes in the circulating CD4^+^ and CD19^+^ T lymphocytes of untreated patients with autoimmune hepatitis have been hypo-methylated, and this pattern has contrasted with the hyper-methylated pattern in PBC (21) (**Table 3**). The predominant hypo-methylated pattern has also been recognized in liver-infiltrating, periportal lymphocytes, and it has been reversible after glucocorticoid-induced, laboratory remission (21). The shift in the pre-treatment pattern of DNA hypo-methylation to the post-treatment pattern of DNA hyper-methylation has occurred in most genes, and it suggests that DNA hypo-methylation promotes disease activity by broadly enhancing the transcriptional activity of multiple genes. The cues that trigger the hypo-methylated state, the hypo-methylated genes that account for active disease, and the glucocorticoid actions that shift the methylation status and achieve remission are unclear.

**Table 3:** Epigenetic marks in autoimmune hepatitis and other autoimmune liver diseases.

Autoimmune Liver Disease	Epigenetic Marks	Epigenetic Effects
Autoimmune hepatitis	Hypo-methylated genes in CD4^+^ T cells (21) Mainly in periportal lymphocytes (21) Shifts to hyper-methylated with steroids (21)	Contrasts with PBC (21) May increase gene transcription (21) May promote disease activity (21) Reversible with steroid treatment (21)
	Serum miR-21 and miR-122 increased (70, 71)	Correlates with inflammation (70) Inversely correlates with fibrosis (70)
	Hepatic miR-122 reduced in cirrhosis (196)	Deficiency promotes fibrosis (196) miR-122 inhibits *P4HA1* in HSCs (197) Deficiency favors collagen formation (198)
	Circulating miR-155 levels low (199) miR-155 increased in liver tissue (199)	Contrasts with ALD and NASH (199) May indicate autoimmune process (199)
PBC	Preferential silencing of X chromosome (200) Excessive silencing Y chromosome (201)	Affects female predisposition (200, 201)
	De-methylation of gene for CXCR3 (59)	Affects hepatic migration of T cells (59)
	H4 acetylation of pro-inflammatory genes (202)	Influences inflammatory activity (202)
	Hypo-methylation of gene for CD40L (203)	Promotes B cells, IgM production (203)
	miR-122, miR-141, miR-26 panel (160) Down-regulation of miR-223 and miR-21 (204)	High diagnostic accuracy for PBC (160) Signals histological progression (204)
PSC	H3K4me3 of *CDKN2A* (63, 205)	Increases cholangiocyte senescence (63) Possible disease progression (63)
	H3K27ac of *BCL2-like 1* (64)	Increases anti-apoptotic BCL-xL (64) Promotes survival of senescent cells (64)

ALD, alcoholic liver disease; BCL2-like 1, B-cell lymphoma 2-like 1 gene; BCL-xL, B-cell lymphoma-extra large; CD40L, CD40 ligand; CDKN2A, cyclin-dependent kinase inhibitor 2A gene; CXCR3, C-X-C chemokine receptor 3; H3K4me3; trimethylation of H3 at lysine 4; H3K27ac, acetylation of H3 at lysine 27; H4, histone 4; HSCs, hepatic stellate cells; IgM, immunoglobulin M; NASH, non-alcoholic steatohepatitis; P4HA1, prolyl-4-hydroxylase subunit alpha-1 gene, PBC, primary biliary cholangitis; PSC, primary sclerosing cholangitis. Numbers in parentheses are references.

### 6.2 MiRNA Profiles

Circulating levels of miR-21 and miR-122 have been increased in untreated patients with type 1 autoimmune hepatitis (70, 71, 196), and miR-155 has been increased in hepatic tissue (71, 196, 199) (**Table 3**). The serum miR-21 and miR-122 levels have correlated with serum alanine aminotransferase (ALT) levels, and the serum miR-21 level has correlated with the histological grade of liver inflammation (70). The histological expression of miR-21 in liver tissue has also correlated with serum ALT levels (196).

In contrast, the serum levels of both miR-21 and miR-122 have correlated inversely with the stage of hepatic fibrosis (70), and reduced hepatic concentrations of miR-122 have been associated with cirrhosis (196) (**Table 3**). MiR-122 markedly attenuates the expression of the gene for prolyl-4-hydroxylase subunit alpha-1 (*P4HA1*) in hepatic stellate cells (197), thereby preventing the hydroxylation and maturation of stable collagen (198). The findings in autoimmune hepatitis suggest that serum miR-21 and miR-122 levels are biomarkers of inflammatory activity (206) and that a pathological deficiency of miR-122 may promote hepatic fibrosis by de-repressing *P4HA1* (196, 197).

Circulating levels of miR-155 have been significantly lower in patients with autoimmune hepatitis regardless of glucocorticoid therapy than in normal individuals (199) (**Table 3**). In contrast, miR-155 concentrations in liver tissue from patients with autoimmune hepatitis have been 7.6 ± 5.6-fold higher than in liver tissue obtained from normal control subjects (*P*< 0.01) and significantly higher than in liver tissue from patients with alcoholic liver disease or non-alcoholic steatohepatitis (NASH) (199). The findings suggest that the hepatic expression of miR-155 in autoimmune hepatitis is particularly associated with immune-mediated liver injury. This possibility is supported by the implication of miR-155 in the pathogenesis of other autoimmune diseases (207). The discrepancy between serum and tissue levels may reflect active mobilization of miR-155 from the circulation to the liver.

### 6.3 Familial Occurrence

The heritability of autoimmune hepatitis through epigenetic traits is unexplored. The familial occurrence of autoimmune hepatitis in Sweden has been mainly among siblings (208, 209) and spouses (208). Among 6269 patients with autoimmune hepatitis in a Swedish database, only siblings have had a significantly increased risk [standardized incidence ratio (SIR), 3.83, 95% confidence interval (CI), 2.09-6.45] (208). Furthermore, the risk for autoimmune hepatitis has been greater among spouses than among siblings (SIR for husbands, 5.91, 95% CI, 2.53-11.7; SIR for wives, 6.07 (95% CI, 2.59-12.02) (208). The risk of autoimmune hepatitis among siblings and spouses in Sweden suggests that epigenetic changes induced by environmental factors may be contributory.

The SIR of autoimmune hepatitis among first-degree relatives has been 4.9 (95% confidence interval [CI], 1.8-10.7) in a Danish database, and the 10-year cumulative risk of autoimmune hepatitis in this group has been 0.10% (95% CI, 0.04-0.23) (210). Among second-degree relatives, there has been no increased risk, whereas among monozygotic twins, the concordance rate for autoimmune hepatitis has been 8.7% (95% CI, 1.1-28) (210). In the composite experience of 32 medical centers in the Netherlands, familial occurrence has been recognized in 0.3% of 564 patients with autoimmune hepatitis, and the disease has occurred in monozygotic twins, the mother of a patient, and the cousin of another patient (211). In each of these experiences, the overall risk of autoimmune hepatitis in family members has been low; heritability has rarely extended beyond the first generation; shared environmental exposures have not been assessed; and the contribution of shared genetic factors has not been evaluated. Differences in the community occurrence of autoimmune hepatitis might also be valuable in assessing non-genetic factors for the disease.

## 7 Epigenetic Changes in Other Autoimmune Liver Diseases

Histone modifications, DNA methylation status, and miRNAs in blood and liver tissue have been evaluated in experimental models and patients with diverse autoimmune and non-autoimmune liver diseases (19, 39, 86). The investigations have been driven by efforts to catalogue the disease-associated findings and identify associations with pivotal pathogenic mechanisms. Key insights have been derived from studies of PBC (212–214) and PSC (63, 64, 212), and they may prompt and direct future investigations of autoimmune hepatitis (19, 21, 70). The epigenetic changes have not been evaluated for disease-specificity nor have they been fully translated into clinical phenotypes.

### 7.1 Epigenetic Findings in PBC

The epigenetic changes described in PBC have been discovered mainly by assessing factors influencing its clinical phenotype (212–214). The importance of epigenetic changes has been demonstrated in monozygotic twins concordant (215) and discordant (216) for PBC, and the female predisposition for PBC has guided investigations of the epigenetic influence on the X-chromosome. A preferential, parent-specific, silencing of the X chromosome has been described in women with PBC (200), and an excessive epigenetic silencing of alleles of the Y chromosome has been demonstrated in men with PBC (201). Furthermore, an aberrant DNA methylation pattern of the promoter region of *CXCR3* on the X chromosome of CD4^+^, CD8^+^, and CD14^+^ T cells may affect their differentiation and hepatic migration (59) (**Table 3**). The acetylation of histone 4 in the promoter region of diverse pro-inflammatory genes can enhance their expression in PBC (202), and DNA hypo-methylation of the gene expressing the CD40 ligand (CD40L, also called CD154) in CD4^+^ T cells may promote B cell maturation and immunoglobulin class switching. The epigenetic effect may contribute to the increased serum levels of immunoglobulin M (IgM) in PBC (203).

A panel of miRNAs, including miR-122-5p, miR-141-3p, and miR-26b-5p, has had high diagnostic accuracy for PBC and a sensitivity that has exceeded that of the serum alkaline phosphatase level (160) (**Table 3**). Step-down expression of miR-223-3p and miR-21-5p in peripheral blood B cells has signaled histological progression of PBC from stage I to stage III (204), and decreased levels of the molecules involved in the biogenesis of miRNAs (prolyl 4-hydroxylase subunit alpha 1 and Argonaute 2) have suggested a widespread disruption of the homeostatic network in a murine model of PBC (61). This hypothesis has been supported by experimental evidence that non-selective stimulation of miRNA biogenesis with enoxacin can up-regulate miRNA production in CD8^+^ T cells, decrease T cell proliferation, and reduce interferon-gamma (IFN-γ) production (61).

### 7.2 Epigenetic Findings in PSC

The epigenetic factors contributing to the progression of PSC have focused mainly on factors influencing the phenotype of the cholangiocytes. Senescent cholangiocytes, defined as cells that have been irreversibly arrested in the G1 or G2 phase of the cell cycle (217, 218), are abundant in the liver of patients with PSC (205) (**Table 3**). The cholangiocytes exhibit features of a senescence-associated secretory phenotype (SASP) that is characterized by the hypersecretion of pro-inflammatory cytokines, chemokines and growth factors (205, 219). The *cyclin-dependent kinase inhibitor 2A (CDKN2A) gene* has been associated with cholangiocyte senescence (205), and histone methylation (H3K4me3) increases its transcriptional activity and the possibility of disease progression (63). Histone acetylation (H3K27ac) of the promoter of the *B-cell lymphoma 2-like 1 gene (BCL2-like 1)* increases expression of the anti-apoptotic protein, B-cell lymphoma-extra large (BCL-xL). This epigenetic change may promote the resistance of senescent cholangiocytes to apoptosis and prolong their survival (64). Both sites of histone modification have been proposed as potential therapeutic targets (63, 64).

The studies in PBC and PSC affirm the strong association of epigenetic modifications in immune-mediated chronic liver disease, and they suggest that the epigenetic modifications can impact on the clinical phenotype, reflect disease-specificity, aid in diagnosis, and direct future therapeutic interventions. They also identify key areas in autoimmune hepatitis that have been unassessed or under-evaluated. Investigations of the epigenetic effects on the X and Y chromosomes, familial predisposition, and heritability of autoimmune hepatitis are wanting.

## 8 Epigenetic Findings in Non-Autoimmune Liver Diseases

Studies in alcoholic liver disease (54, 55) and non-alcoholic fatty liver disease (NAFLD) (220–229) have emphasized the pervasive, interactive, and composite effects of epigenetic modifications in each disease. They have also indicated the needs to associate changes in disease expression to clinically relevant features and to explore the heritable and adaptive nature of the epigenetic modifications. These insights are foundational for future studies in autoimmune hepatitis since they may clarify the mechanisms of occurrence, recurrence and progression.

### 8.1 Epigenetic Findings in Alcoholic Liver Disease

A plethora of epigenetic changes involving DNA methylation, histone modification, and circulating miRNA levels have been described in experimental models and patients with alcoholic liver disease and alcoholic steatohepatitis (54, 55). Epigenetic modifications have been demonstrated in genes that may influence the metabolism of ethanol (230–234), the activity of enzymes that mediate histone acetylation (HATs, sirtuins) (55, 235), the vigor of the inflammatory response (236), and the generation of hepatic fibrosis (224, 237) (**Table 4**). Furthermore, increased circulating levels of several miRNAs have been described that may be biomarkers of alcohol-related liver injury (miR-155) (55, 238, 241, 242), indicators of a disrupted intestinal mucosal barrier (miR-212) (239), or mediators of lipid and cholesterol metabolism (miR-122) (240). The abundance of epigenetic changes and interactions has indicated a complexity that must be edited for clinical relevance. A similar complexity of epigenetic interactions can be anticipated in autoimmune hepatitis, and future investigations must be directed by the pivotal clinical needs to understand and control the severity, progression, and recurrence of the disease.

**Table 4:** Epigenetic marks in non-autoimmune liver diseases.

Non-Autoimmune Liver Disease	Epigenetic Marks	Epigenetic Effects
Alcoholic liver disease	DNA methylation changes (54, 55) Histone PTMs (54, 55) Multiple genes affected (54, 55)	Modulation of ethanol metabolism (234) Mediation of inflammation (236) Progression of liver fibrosis (224, 237) Activity of histone acetylation (55, 235)
	Increased circulating miR-155 (238) Increased circulating miR-212 (239) Increased circulating miR-122 (240)	Biomarker of alcohol injury (55, 241, 242) Denotes disrupted intestinal barrier (239) Mediates lipid metabolism (240)
NAFLD	Hyper-methylated *PNPLA3* (220) Hypo-methylated *PARVB1* (220) Variably DNA methylated genes (224) Histone acetylation of *TNFA* (225) Histone acetylation of *FASN* (226)	Hepatic steatosis and inflammation (221, 222) Hepatic fibrosis, steatosis, activity score (223) Severe hepatic fibrosis (224) Increased inflammation (225) Up-regulated lipogenesis in hepatocytes (226)
	Increased circulating miR-122, miR-34a, and miR-16 (227)	miR-122, miR-34a associated with lipid levels, fibrosis stage, and inflammation (227)
	High serum miR-122 levels (228) Low liver miR-122 levels (228, 229)	Increased serum ALT activity (228) Associated with NASH (228, 229)
	mir-331-3p and miR-30c strongly associated with each other (194) Present in twins with NAFLD (194) May be heritable (194)	Lipid and metabolic pathways (194)

ALT, alanine aminotransferase; DNA, deoxyribonucleic acid; FASN, fatty acid synthase gene; NAFLD, non-alcoholic fatty liver disease; NASH, non-alcoholic steatohepatitis; PARVB1, parvin beta 1 gene; PNPLA3, patatin-like phospholipase domain-containing protein 3 gene; PTMs, post-translational modifications; TNFA, tumor necrosis factor alpha gene. Numbers in parentheses are references.

### 8.2 Epigenetic Findings in NAFLD

Hypermethylation of CpG99 in the regulatory region of the *patatin-like phospholipase domain-containing protein 3 (PNPLA3) gene* and hypomethylation of CpG26 in the regulatory region of the *parvin beta 1 (PARVB1) gene* have been associated with advanced hepatic fibrosis in patients with NAFLD (220) (**Table 4**). The rs738409 (G-allele) polymorphism of *PNPLAS* has been associated with hepatic steatosis and inflammation in patients with NAFLD (221, 222), and the *PARVB* variant has been associated with steatosis grade, NAFLD activity score, and hepatic fibrosis (223). Differential methylation of CpG sites within other genes known to affect hepatic fibrosis have also distinguished patients with severe fibrosis (224).

Histone acetylation of the pro-inflammatory genes, *tumor necrosis factor alpha* (*TNFA*) and *monocyte chemotactic protein 1* (*MCP1*; also called *CD2*) have been up-regulated in a murine model of obesity (225), and the histone acetylation of the gene stimulating transcription of fatty acid synthase (*FASN*) has been associated with *de novo* lipogenesis in human hepatocytes (226) (**Table 4**). Serum levels of miR-122, miR-34a and miR-16 have also been increased in patients with NAFLD compared to patients with chronic hepatitis C, and the serum levels of miR-122 and miR-34a have correlated with biochemical tests and histological assessments of fibrosis stage and inflammatory activity (227).

Serum levels of miR-122 have also been 7.2-fold higher in patients with non-alcoholic steatohepatitis (NASH) than in healthy control subjects and 3.1-fold higher in patients with NASH than in patients with simple steatosis (228) (**Table 4**). Hepatic expression of miR-122 has been down-regulated in NASH compared to patients with simple steatosis (228) or normal liver (229), and the hepatic expression of miR-122 has been mostly near lipid-laden hepatocytes (228). The physiological significance of miR-122 in the development of NASH has been postulated, but not evident in all investigations (243) or validated as a pivotal pathogenic factor (194).

Studies of monozygotic and dizygotic twins have demonstrated that discordance for NAFLD has been associated with 21 miRNAs, including miR-122 (*P*=0.002) and miR-34a (*P*=0.04) (194) (**Table 4**). MiR-331-3p (*P*=0.0007) and miR-30c (*P*=0.011) have been preferentially expressed in the twins with NAFLD, and the strong correlation of miR-331-3p and miR-30c with each other (R=0.90, *P*=2.2 x 10^-16^) has suggested their shared involvement in NAFLD (194). This hypothesis has been supported by evidence that the seven gene targets shared by miR-331-3p and miR-30c have included genes affecting lipid and metabolic pathways (194).

The multiplicity of epigenetic changes associated with NAFLD may reflect differences in environmental cues (lifestyle, diet, age-related exposures, surgeries) (184, 187, 193) and transgenerational inheritance of gene modifiers (25, 38, 194). The profiling of the epigenome of sperm from lean and obese men has disclosed marked differences in the expression of small non-coding RNA and DNA methylation patterns which may have reflected inherited and acquired changes (184) (**Table 4**). The rapid remodeling of DNA methylation in the sperm of morbidly obese men who have undergone bariatric surgery has indicated the dynamic plasticity of epigenetic changes under environmental pressure (184). The challenge has been to identify the key factor or combination of factors that can be moderated in a particular clinical situation. The plasticity of the epigenetic changes in response to environmental cues or therapeutic intervention and the expression of these epigenetic responses in the germline are key features that warrant investigation in autoimmune hepatitis.

## 9 Epigenetic Manipulations

Therapeutic manipulation of disease-associated epigenetic changes is possible by interventions that affect the enzymes that modify the chromatin structure, the targets recognized by circulating miRNAs, and the environmental factors that promote instability of the epigenome (39, 86, 244). Interventions that affect enzymatic modulation of the chromatin structure have been directed at DNA methylation (245), histone methylation (246, 247), and histone acetylation (248–250). Interventions that affect target recognition by pivotal miRNAs have involved engineered molecules that mask the chosen gene product or substitute a decoy (251–256). Interventions that stabilize the epigenome have included risk-reduction, lifestyle modifications (19, 34, 257) and dietary supplementation with S-adenosylmethionine (258, 259), methyl group donors (260, 261), vitamin C (122), or vitamin D (262–267). The major concerns have been the lack of target selectivity and the uncertain risk of deleterious off-target consequences (19, 43).

### 9.1 Therapeutic Modulation of Chromatin Structure

DNMT inhibitors, HDAC inhibitors, HDAC activators, and HMT inhibitors have been the principal interventions directed at the enzymatic bases for disease-associated epigenetic changes in chromatin. These interventions have been studied mainly in experimental models of liver disease and patients with malignancy (39, 86, 244) (**Table 5**).

**Table 5:** Therapeutic manipulations of epigenome.

Enzymes	Rationale	Experimental and Clinical Experience
DNMT inhibitors	DNA hyper-methylation in HCC (245)	Guadecitabine limits HCC in mice (245)
HDAC inhibitors	HDACs high in HBV-related HCC (268) HDAC inhibitors tolerated in trials (269)	HDAC inhibitors limit HCC *in vitro* (268) Panobinostat effective in HCC model (249)
HDAC activators	Deficient sirtuin 1 in NAFLD model (270) Resveratrol activates sirtuin 1 (271–274)	Resveratrol limits rodent NAFLD (272) Resveratrol no effect in humans (250)
HMT inhibitors	EZH2 catalyzes histone methylation (275) H3K27me3 represses *PPARG* (276) *PPARG* inhibition increases fibrosis (277)	DZNep inhibits liver fibrosis in mice (247)
**miRNA targets**		
Anti-sense oligonucleotides	Limits miRNA binding to mRNA (278) Preserves mRNA (71, 279)	Clinical trials in diverse diseases (280–282)
Decoy mRNA targets	Decoy mRNA binds miRNA (254–256) “Sponge effect” depletes miRNA (255)	Experimental models (254–256)
Drugs	General miRNA deficiency possible (61) Drugs can enhance miRNA biogenesis (61)	Enoxacin down-regulated CTLs in murine PBC (61)
**Supplements**		
SAM	Methyl groups improve methylation (259) Dietary methyl groups helped in rats (261)	Less demethylase activity in cell lines (258) Preserved DNA methylation (259)
Vitamin C	Supports activity of TET enzymes (122)	De-methylated DNA in mouse cells (122)
Vitamin D	Limits transcription of TGF-β, TIMP (266)	Prevents experimental fibrosis (262–265)

CTLs, cytotoxic CD8^+^ T cells; DNA, deoxyribonucleic acid; DNMT, DNA methyltransferase; DZNep, 3-deazaneplanocin A; EZH2, enhancer of zeste homolog 2; HBV, hepatitis B virus; HCC, hepatocellular carcinoma; HDAC, histone deacetylases; HMT, histone methyltransferase; mRNA, messenger ribonucleic acid; miRNA, micro-ribonucleic acid; NAFLD, non-alcoholic fatty liver disease; PPARG, peroxisome proliferator-activated receptor gamma gene; PBC, primary biliary cholangitis; SAM, S-adenosylmethionine; TGF-β, transforming growth factor-beta; TET, ten-eleven translocation enzyme; TIMP, tissue inhibitors of metalloproteinases. Numbers in parentheses are references.

#### 9.1.1 DNA Methyltransferase Inhibition

DNA hyper-methylation has been a strong feature of hepatocellular carcinoma (HCC), and guadecitabine (also called SGI-110) is a DNMT inhibitor. Guadecitabine has sensitized HCC cells to oxaliplatin by inhibiting signaling pathways that have promoted HCC growth in mice (245).

#### 9.1.2 Histone Deacetylase Inhibition

HDACs have been highly expressed in patients with HCC related to chronic hepatitis B virus infection, and they have been a prognostic biomarker associated with tumor growth and reduced survival (268). HDAC inhibition has suppressed proliferation of HCC cells *in vitro* (268), and the pan-HDCA inhibitor, panobinostat, has been effective in experimental models of HCC when combined with sorafenib (249). HDAC inhibitors have been well-tolerated in clinical protocols, and trials have been extended to non-tumorous diseases, including neurodegenerative diseases and inflammatory disorders (269) (**Table 5**).

#### 9.1.3 Histone Deacetylase Activation

Sirtuin 1 (SIRT1) promotes the deacetylation of histones and regulates glucose and fat metabolism (270, 283). Deficient hepatic expression of SIRT1 has been accompanied by metabolic dysfunction in a murine model (270). The polyphenol, resveratrol, activates the deacetylase, SIRT1 (248, 284, 285), and it has improved the survival of mice on a high calorie diet (286) (**Table 5**). Resveratrol has also protected rodents from diet-induced steatohepatitis through a variety of signaling pathways (271–274). Resveratrol has not had a therapeutic benefit in overweight and obese men with established NAFLD (250), and the role of HDAC activation as a protective or therapeutic intervention for NAFLD remains unclear in humans.

#### 9.1.4 Histone Methyltransferase Inhibition

Epigenetic modifications of chromatin have been implicated in the trans-differentiation of hepatic stellate cells into myofibroblasts (39), and the enzymes that regulate the methylation of DNA (287, 288) and histone (246) have been prime therapeutic targets (247, 277). Hepatic fibrosis is regulated by a series of epigenetic relays that include down-regulation of miR-132, binding of the methyl-CpG binding protein 2 (MeCP2) to the 5’ end of the PPAR-γ-producing gene (*PPARG*), and activation of the enhancer of zeste homolog 2 (EZH2) (277) (**Table 5**).

EZH2 is an epigenetic regulator that represses gene transcription by catalyzing the trimethylation of histone 3 at lysine 27 (H3K27me3) (275, 289, 290). The formation of H3K27me3 in the 3’ exon of *PPARG* represses the anti-fibrotic effect of this gene (276) and promotes hepatic fibrosis (277) (**Table 5**). Therapeutic disruption of the pro-fibrotic epigenetic pathway is possible at multiple sites, but the pivotal epigenetic step for myofibroblast differentiation is trimethylation of *PPARG* at H3K27 (276). 3-Deazaneplanocin A (DZNep) is a pan-inhibitor of histone methyltransferase, and its use in a murine model of toxin-induced liver injury has inhibited the histological progression of hepatic fibrosis (247).

### 9.2 Therapeutic Modulation of MiRNAs

MiRNAs are prime targets for therapeutic manipulation because circulating miRNA levels have distinguished certain diseases and the gene silencing action of miRNAs can disrupt pivotal homeostatic pathways that regulate immune and inflammatory responses (40, 42, 43). The principal method of targeting miRNAs in experimental models and patients in clinical trials has been the use of anti-sense oligonucleotides (antimirs) (71, 278, 279) (**Table 5**). These molecules are engineered to block the binding of a selected miRNA to its targeted mRNA, and they prevent the miRNA from silencing the gene product. The binding affinity, stability, and potency of antimirs can be enhanced by diverse modifications of the core molecule. The modified molecules have been designated antagomirs (252, 253, 291). Anti-sense obligonucleotides have been evaluated in clinical treatment trials for Alport syndrome (280), chronic hepatitis C (281), and chronic lymphocytic leukemia (282).

RNA transcripts have also been designed to mimic the selected natural mRNA and protect it from degradation or translational repression by miRNAs. The decoy mRNA binds with the natural miRNA and prevents it from silencing the natural mRNA (254–256) (**Table 5**). Drugs have also been used to non-selectively stimulate the biogenesis of miRNAs (61). Widespread deficiency of miRNAs may allow the expression of genes that promote disease activity, and drug-induced, non-selective stimulation of miRNA biogenesis may silence the expression of these deleterious genes (61). These interventions await rigorous preclinical evaluations and clarification of their safety profile (43).

### 9.3 Therapeutic Modulation of Environmental Factors and Use of Dietary Supplements

Multiple environmental factors have been associated with diverse epigenetic changes, and lifestyle modifications may reduce the risk of disease-provoking epigenetic changes (19, 34, 58, 257, 292, 293) (**Table 5**). Medications (procainamide, hydralazine, and 5-azacytidine) (294–297), pollutants (tobacco smoke, aerosolized contaminants, and heavy metals) (298–301), and infection (302) have been associated with changes in DNA methylation that may affect gene expression. Furthermore, environmentally-induced epigenetic changes have been associated with the occurrence or progression of diverse immune-mediated diseases (rheumatoid arthritis, PBC, and SLE) (72, 298, 303–305). Epigenetic changes that are potentially deleterious and heritable have also been associated with nutritional deficiencies, stress, ultraviolet light, radiation, and trauma (19, 34, 257). Lifestyle modifications that avoid excessive, high risk exposures may protect against deleterious epigenetic effects, but their efficacy has been difficult to establish.

Dietary supplements have also been described in experimental animals that enhance the supply of methyl groups (S-adenosylmethionine, diverse methyl donors) (258–261), activate the TET enzymes that de-methylate DNA (vitamin C) (122), and alter the transcription of mRNAs that promote hepatic fibrosis (vitamin D) (262–267) (**Table 5**). S-adenosylmethionine has inhibited demethylase activity and preserved DNA methylation in cell lines (258, 259). Dietary supplementation with methyl groups has promoted DNA hyper-methylation and prevented transgenerational amplification of obesity in a mouse model (260). It has also modified the methylation profile of the gene expressing fatty acid synthase and reduced hepatic triglyceride accumulation in rats fed a high fat, high sucrose diet (261). Vitamin C has supported the activity of TET enzymes, and it has promoted the de-methylation of DNA in the embryonic stem cells of mice (122). 1, 25-dihydroxyvitamin D has repressed the transcription of mRNAs for TGF-β and tissue inhibitors of metalloproteinases (TIMP). It has also up-regulated the transcription of metalloproteinases and prevented progressive hepatic fibrosis (262–267). These promising pre-clinical experiences await validation in randomized clinical trials that define their utility in specific diseases (259).

## 10 Epigenetic Prospects in Autoimmune Hepatitis

Findings that have already been made in diverse autoimmune (63, 64, 212–214) and non-autoimmune (220, 223–226, 232) liver diseases support the prospect that multiple, clinically-relevant, epigenetic marks will be identified in autoimmune hepatitis. They also support the prospect that pivotal genes affecting critical pathogenic pathways will be recognized and that interventions will be assessed to modify an aberrant gene expression or pattern (247, 250, 260, 261, 271, 281). The success of these projections in changing the management of autoimmune hepatitis will depend on proofs of causality, confident identification of critical gene expressions or patterns, and precise editing of the epigenetic landscape.

### 10.1 Proofs of Causality

Progress toward targeted epigenetic management of autoimmune hepatitis ideally requires proof of causality for each epigenetic mark and a hierarchy of candidates based on measured consequences. Methods that disrupt and restore the epigenetic mark in experimental models or cell systems can establish and quantify causality. The clustered, regularly interspaced, short palindromic repeats (CRISPR) of base sequences in segmental DNA and the CRISPR-associated protein 9 (Cas9) system consists of a guide RNA that matches the DNA target site and an endonuclease (Cas9) that performs site-specific DNA cleavage (306, 307) (**Table 6**). This system has been re-purposed for epigenetic editing by engineering a “deactivated” Cas9 protein (dCas9) that lacks nuclease activity (309–312, 320, 321). The CRISPR-dCas9 system can target specific DNA loci without changing the DNA sequence, and dCas9 can deliver sequence-specific motifs to a desired location in the epigenome (308). Site-specific epigenetic editing that can block or restore gene expression in experimental models or cell lines can prove causality and develop a hierarchy of candidates for therapeutic targeting.

**Table 6:** Epigenetic prospects in autoimmune hepatitis.

Epigenetic Prospects	Rationale	Expectations
Proofs of causality	Epigenetic marks lack proofs of causality (308) CRISPR-dCas9 allows epigenetic editing (309) Site-specific editing can prove causality (308) Hierarchy of targets possible (310–312)	Prime therapeutic target(s) selected (308)
Identification of gene targets or patterns	miR-21 is cue to affected genes (70)	Key gene prospects of miR-21 assessed: • *programmed cell death protein 4* (313) • *TNF-α-induced protein 8-like 2* (314)
	miR-122 is cue to affected genes (70, 196)	Key gene prospects of miR-122 assessed: • *hypoxia inducible factor 1-α* (315) • *prolyl-4-hydroxylase subunit α-1* (197)
	miR-155 is cue to affected genes (199, 207)	Key gene prospects for miR-155 assessed: • *suppressor of cytokine signaling* (316) • *c-musculoaponeurotic fibrosarcoma* (317) • *Src homology 2-containing inositol-5’-phosphatase 1* (318)
	Hypo-methylated genes already recognized (21)	Key hypo-methylated prospect assessed: • *forkhead box p3 (Foxp3)* (319)
	Multiple genes can have composite effect (21) Gene patterns can affect outcome (194)	Epigenetic network recognized (19)
Therapeutic epigenetic editing	CRISPR-dCas9 edits precisely (309–312, 320, 321)	Individual and multiple edits possible (312) Elimination of non-selective enzymes (308) Site-specific enzyme delivery (308, 312) Correction of miRNA deficiencies Modulation of miRNA gene expression Homeostasis of stimulatory/inhibitory genes
	Uncertain off-target effects (43)	Highly selective, precise edits (308, 312) Rigorous safety assessments Monitoring protocols

CRISPR, clustered, regularly interspaced, short palindromic repeats; dCas9, deactivated CRISPR-associated protein 9; miRNA, micro-ribonucleic acid; TNF, tumor necrosis factor. Numbers in parentheses are references.

### 10.2 Identification of Critical Gene Targets or Patterns

A distinctive profile of circulating miRNAs (70, 71, 196, 199) and the hypo-methylation of multiple genes (21) have already been described in autoimmune hepatitis. Future investigations must identify the genes whose expressions are affected by these miRNAs (miR-21, miR-122, and miR-155) and the hypo-methylation (**Table 6**). The hypo-methylated *forkhead box p3 (Foxp3) gene* stabilizes the expression of Foxp3 on regulatory T cells (Tregs) and maintains their integrity (319). Preservation of this hypo-methylated state may constitute a mechanism by which to achieve and maintain quiescent disease (322, 323). Hypo-methylation may also stimulate genes with deleterious actions, and treatments that hyper-methylate genes non-selectively may compromise Treg function (322). Clarification of the genes implicated in autoimmune hepatitis by the circulating miRNAs and their hypo-methylated status will be essential in understanding the complexity and interactivity of potential epigenetic targets.

Additional characterization of the epigenome of autoimmune hepatitis can be anticipated, and it may identify multiple up- and down-regulated genes that have a composite effect. Multiple gene expressions have distinguished patients with NAFLD (194), and multiple hypo-methylated genes have been described in autoimmune hepatitis (21). The multiplicity of implicated genes may reveal a pattern that distinguishes autoimmune hepatitis and influences its phenotype and outcome. The pattern may also reveal a common basis for autoimmunity or have disease-specificity.

### 10.3 Editing the Epigenetic Landscape

The CRISPR-dCas9 system promises to replace the use of enzymes that non-selectively alter DNA methylation and PTMs (309–312, 320, 321) (**Table 6**). It may also limit or eliminate the need to target miRNAs with anti-sense oligonucleotides (71, 252, 278, 279) or mRNA mimics (254–256). HATs, acetyl groups, DNMTs, and TET enzymes can be tethered to the dCas9 protein and delivered to the chosen epigenetic site by the CRISPR-dCas9 system (312, 320). The effectiveness of the CRISPR-dCas9 system in editing the epigenome of autoimmune diseases is unknown, but future investigations should evaluate its ability to edit multiple epigenetic marks, restore homeostatic balance between immune stimulatory and inhibitory genes, and modulate the genes that generate particular miRNAs. The major safety concern is the uncertainty of unintended off-target effects (34, 43).

## 11 Conclusions

The epigenome is a largely unstudied domain in autoimmune hepatitis, and its rigorous evaluation may yield results that complement, complete, or change the current knowledge base. The epigenome is dynamic, reactive, adaptable, reversible, and potentially heritable. The epigenetic landscape could influence the predisposition, phenotype, pathogenesis, and outcome of autoimmune hepatitis, and it could reflect environmental factors that can be modified or avoided. The epigenetic landscape could also have diagnostic and prognostic implications that could help direct management. Methods that allow highly selective editing of the epigenome promise to expand treatment options by modulating the expression of pivotal genes or the composite effect of multiple genes. The key challenges are to determine the pivotal epigenetic changes or patterns associated with autoimmune hepatitis, understand the interactive network of genes with opposing actions that promote the disease, and develop interventions that restore homeostatic balance with minimal risk of unintended off-target consequences.

## Author Contributions

AC researched, designed, and wrote this article. The tables and figure are original, constructed by the author, and developed solely for this review.

## Conflict of Interest

The author declares that the research was conducted in the absence of any commercial or financial relationships that could be construed as a potential conflict of interest.

The handling editor NK declared a past co-authorship with the author.

## Publisher’s Note

All claims expressed in this article are solely those of the authors and do not necessarily represent those of their affiliated organizations, or those of the publisher, the editors and the reviewers. Any product that may be evaluated in this article, or claim that may be made by its manufacturer, is not guaranteed or endorsed by the publisher.
